# ACE2 inhibits breast cancer angiogenesis via suppressing the VEGFa/VEGFR2/ERK pathway

**DOI:** 10.1186/s13046-019-1156-5

**Published:** 2019-04-25

**Authors:** Qi Zhang, Sihong Lu, Tianfu Li, Liang Yu, Yunjian Zhang, Huijuan Zeng, Xueke Qian, Jiong Bi, Ying Lin

**Affiliations:** 1 Breast Disease Center, The First Affiliated Hospital, Sun Yat-Sen University, No.58 of Zhongshan 2nd road, Yuexiu district, Guangzhou, 510080 China; 2 Laboratory of Surgery, The First Affiliated Hospital, Sun Yat-Sen University, No. 58 of Zhongshan 2nd road, Yuexiu district, Guangzhou, 510080 China; 3 Guangdong Key Engineering Laboratory for Diagnosis and Treatment of Vascular Disease, The First Affiliated Hospital, Sun Yat-Sen University, Guangzhou, 510080 China; 4 The First Affiliated Hospital, Zhengzhou University, Zhengzhou, 450000 China

**Keywords:** Breast cancer, Angiogenesis, ACE2, VEGFa, VEGFR2, ERK

## Abstract

**Background:**

Breast cancer angiogenesis is key for metastasis and predicts a poor prognosis. Angiotensin-converting enzyme 2 (ACE2), as a member of the renin-angiotensin system (RAS), was reported to restrain the progression of hepatocellular carcinoma (HCC) and non-small cell lung cancer (NSCLC) through inhibiting angiogenesis. However, the relationship between ACE2 and breast cancer angiogenesis remains unclear.

**Methods:**

The prognosis and relative gene selection were analysed using the GEPIA, GEO, TCGA and STRING databases. ACE2 expression in breast cancer tissue was estimated by reverse transcription-quantitative polymerase chain reaction (qPCR). Breast cancer cell migration, proliferation and angiogenesis were assessed by Transwell migration, proliferation, tube formation, and wound healing assays. The expression of vascular endothelial growth factor A (VEGFa) was detected by qPCR and Western blotting. The phosphorylation of vascular endothelial growth factor receptor 2 (VEGFR2), mitogen-activated protein kinase 1/2 (MEK1/2), and extracellular signal-regulated protein kinase 1/2 (ERK1/2) was examined by Western blotting. Breast cancer metastasis and angiogenesis in vivo were measured using a zebrafish model.

**Results:**

ACE2 was downregulated in breast cancer patients. Patients with higher ACE2 expression had longer relapse-free survival (RFS). In vitro, ACE2 inhibited breast cancer migration. Meanwhile, ACE2 in breast cancer cells inhibited human umbilical vascular endothelial cell (HUVEC) proliferation, tube formation and migration. In the zebrafish model, ACE2 inhibited breast cancer cell metastasis, as demonstrated by analyses of the number of disseminated foci and the metastatic distance. Neo-angiogenesis was also decreased by ACE2. ACE2 downregulated the expression of VEGFa in breast cancer cells. Furthermore, ACE2 in breast cancer cells inactivated the phosphorylation of VEGFR2, MEK1/2, and ERK1/2 in HUVECs.

**Conclusions:**

Our findings suggest that ACE2, as a potential resister to breast cancer, might inhibit breast cancer angiogenesis through the VEGFa/VEGFR2/ERK pathway.

**Trial registration:**

Retrospectively registered.

**Electronic supplementary material:**

The online version of this article (10.1186/s13046-019-1156-5) contains supplementary material, which is available to authorized users.

## Background

Tumour angiogenesis refers to the formation of new blood vessels in solid tumours for the supply of nutrients and oxygen, hence promoting the spread of tumour cells [1, 2]. It occurs during tumour development and progression and is a key step in tumour metastasis [3, 4]. The vasculature network around the tumour starts to be actively growing and infiltrative in response to the secretion of pro-angiogenetic factors by the tumour, and this process is called the angiogenic switch [5, 6]. The new formed tumour vessels are often twisty, rugged and leaky with discontinuous endothelial cell lining and deficient basement membrane and pericyte coverage. These aberrant morphologies lead to impaired vascular maturation, poor vessel functionality and incoherent tumour perfusion [5, 7, 8]. In addition, various tumour-associated stromal cells and the extracellular matrix constitute the tumour environment to sustain angiogenesis during tumour progression [9, 10]. In breast cancer, the microvessel density (MVD) is a pivotal risk factor for metastasis and a predictor of poor prognosis [11–13]. Thus, breast cancer angiogenesis is a promising diagnostic and therapeutic target that should be investigated [14, 15].

As is well accepted, among the pro-angiogenic factors, vascular endothelial growth factor (VEGF) is the major regulator inducing the sprouting and proliferation of endothelial cells [16] while increasing the permeability of vessels [17, 18]. The secretion of VEGF by tumour cells contributes to neovascularization, which in turn helps the generation and development of cancer [19, 20]. The most functional form of VEGF among its isoforms is VEGFa, which exerts its angiogenic effects by activating VEGFR2 expressed on endothelial cells [21]. Anti-angiogenetic drugs targeting the VEGF pathway are currently used in tumour therapy, particularly in non-small cell lung cancer (NSCLC) [22]. However, these drugs do not exert significant therapeutic effects in breast cancer patients. New targets of anti-angiogenetic therapy need to be found.

Angiotensin-converting enzyme 2 (ACE2), a member of the renin-angiotensin system (RAS), plays an important role in the cardiovascular system by converting angiotensin I (AngI) to Ang(1–9) [23] and converting AngII to Ang(1–7) [24, 25]. The products of this enzyme have effects on vasodilation, anti-proliferation and anti-fibrosis [26, 27]. Recently, researchers have found that members of the RAS participate in different biological processes in various tumours. AngII was reported to facilitate tumour migration, proliferation, angiogenesis and metastasis by activating AngII type 1 receptor (AT1R) [28–30], while the activation of AngII type 2 receptor (AT2R) promotes tumour proliferation and angiogenesis in lung cancer [31, 32]. ACE2, as well as Ang(1–7), was reported to inhibit the growth of lung cancer [33–35] and the metastasis of prostate cancer [36], while it indicates better prognosis in hepatocellular carcinoma [37]. However, the effects of ACE2 on breast cancer angiogenesis remain unknown.

In the present study, we analysed the role of ACE2 in breast cancer prognosis using databases and human tissues, verified the anti-angiogenetic effects of ACE2 in vitro and in vivo and then revealed the VEGFa/VEGFR2/ERK pathway.

## Methods

### Cell culture

Human breast cancer cell lines (MDA-MB-231, MCF-7, BT-549, ZR-75-30, ZR-75-1, MDA-MB-435, BT-474, MDA-MB-468, and T47D) were purchased from the American Type Culture Collection (ATCC; Manassas, VA, USA) and were maintained in Dulbecco’s modified Eagle’s medium (DMEM; Gibco, USA) supplemented with 10% foetal bovine serum (FBS; Gibco), 1% penicillin and streptomycin (Gibco).

Human umbilical vascular endothelial cells (HUVECs) were isolated from the umbilical cord and propagated in endothelial cell medium (ECM; ScienCell, USA) supplemented with 10% FBS (ScienCell), 1% endothelial cell growth supplement (ScienCell), and 1% penicillin and streptomycin (ScienCell).

All the cells were cultured in a humidified incubator at 37 °C with 5% CO_2_.

### Cell transfection

MDA-MB-231 cells overexpressing ACE2 (231-lenti-ACE2) and the matched control cells (231-lenti-Vec) were established using lentivirus carrying the pCMV-ACE2-EGFP-puro plasmid (GeneChem, Shanghai, China). Puromycin (2 μg/ml) was used to select the cells with stable overexpression of ACE2.

MCF-7 cells were transfected with small interfering RNA (siRNA; GenePharma, Shanghai, China) to knock down ACE2 using Lipofectamine RNAiMAX (Invitrogen).

### Tube formation assay

The breast cancer cell culture medium was changed to serum-free DMEM medium for 48 h and then was collected, centrifuged and filtered to obtain tumour-conditioned medium (TCM).

The pipet tips and 96-well plates were precooled, and growth-factor-reduced Matrigel (BD, Corning, USA) was thawed overnight prior to the assay. The wells of the 96-well plate were coated with 50 μl of Matrigel and incubated for 30 min. HUVECs were starved in ECM without serum for 24 h prior to the assay, and 2 × 10^4^ HUVECs were seeded on the gel with 200 μl of TCM concentrated 75-fold using an ultrafiltration device (Millipore, USA). The TCM from MCF-7 cells was supplemented with 1% FBS. The tube formation of HUVECs was observed at different time points during a 12-h experimental period using a microscope.

### Cell migration assay

For the tumour cell Transwell migration assay, transfected breast cancer cells were seeded in DMEM without FBS in the upper chamber. The lower chamber was filled with DMEM containing 10% FBS. After 24 h (MDA-MB-231 cells) or 48 h (MCF-7 cells), the upper chamber was washed, fixed, dyed and photographed.

For the HUVEC wound healing assay, HUVECs were cultured to 100% confluence, and vertical scratches were made using a pipet tip. Next, the medium was replaced with TCM. The wounds were photographed every 24 h for 3 days, and the migration distance of the HUVECs was measured.

### Cell proliferation assay

Transfected breast cancer cells and HUVECs were seeded in 96-well plates. Once the cells were adherent, the culture medium was replaced with TCM. The CCK8 (Dojindo Laboratories, Japan) assay was then conducted according to the manufacturer’s instructions, and the absorbance at a wavelength of 450 nm was read using a microplate reader (Sunrise, Tecan, Austria) every day at the same time for 5 days.

### Reverse transcription-quantitative polymerase chain reaction (qPCR)

The total RNA from human breast tissues or cell lines was extracted using the TRIzol reagent (Invitrogen) and subjected to reverse transcription using the PrimeScript RT reagent Kit (Takara, Japan) to synthesize cDNA according to the manufacturer’s instructions. The qPCR assay was performed using the LightCycler480 system (Roche, Switzerland) and SYBR Green (Takara). The primer sequences are presented in the Additional file 1: Supporting methods.

### Western blotting

The total protein from the cell lines was extracted using RIPA lysis buffer (Beyotime, Shanghai, China) with 1% PMSF (Beyotime), and the concentration was measured using the BCA kit (Beyotime) according to the manufacturer’s instructions. The proteins were separated by SDS-PAGE gel electrophoresis and transferred to polyvinylidene fluoride film (PVDF; Millipore). The film was blocked and incubated with different primary antibodies (at the dilutions shown in Additional file 1: Supporting methods) and the IgG-hydrogen peroxide (HRP) secondary antibody. The protein blots were exposed to ECL luminol reagent (Millipore) using the Amersham Imager 600 Image system (AI600, USA).

### Zebrafish model

Transfected breast cancer cells labelled with the fluorescent carbocyanine dye Dil (orange colour; GeneCopoeia, USA) were microinjected into the perivitelline cavity of tricaine-anaesthetized Tg (fli1: EGFP) zebrafish embryos at 48 h post-fertilization. The embryos were cultivated in Hank’s buffer with 1-phenyl-2-thiourea (PTU) to inhibit pigmentation. The migration of tumour cells in the zebrafish at 0 and 24 h after injection was observed by fluorescence microscopy (Zeiss, German). The vascular structure surrounding the tumour site was photographed using a confocal microscope (LSM780; Zeiss, German).

### Human breast cancer sample

All the breast cancer and normal tissues were obtained from patients diagnosed with breast cancer who received breast-conserving or radical surgery without any neoadjuvant therapy at the First Affiliated Hospital of Sun Yat-sen University between 2017 and 2018. The usage of human tissues was approved by the ethics committee of the First Affiliated Hospital of Sun Yat-sen University.

### Statistical analysis

The expression analysis was performed using Gene Expression Profiling Interactive Analysis (GEPIA) [38, 39]. The Kaplan-Meier survival analysis was conducted using Kaplan-Meier plotter [40, 41] in the Gene Expression Omnibus (GEO) database and the following datasets: E-MTAB-365 GSE11121, GSE12093, GSE12276, GSE1456, GSE16391, GSE16446, GSE16716, GSE17705, GSE17907, GSE19615, GSE20271, GSE2034, GSE20685, GSE20711, GSE21653, GSE2603, GSE26971, GSE2990, GSE31519, GSE3494, GSE37946, GSE42568, GSE45255, GSE4611, GSE4922, GSE5327, GSE6532, GSE7390, and GSE9195. Gene Ontology (GO) enrichment was analysed to explore the function of ACE2 and related genes. The Cancer Genome Atlas (TCGA) was employed to obtain the heat map of ACE2. The String database and the Cytoscape tool cytoHubba were used to calculate the rank of hub genes. The Kyoto Encyclopedia of Genes and Genomes (KEGG) was used for the pathway analysis. All the bioinformatics analyses were performed using the R2: Genomics Analysis and Visualization Platform [42–44].

All the experiments were repeated three times. The statistical significance was analysed by two-tailed Student’s t-test using Prism GraphPad 7.0 software and SPSS 24.0 software, and *P < 0.05 was assumed to indicate statistical significance.

## Results

### ACE2 is downregulated in breast cancer tissue, and this downregulation is associated with worse prognosis

**Fig. 1:**
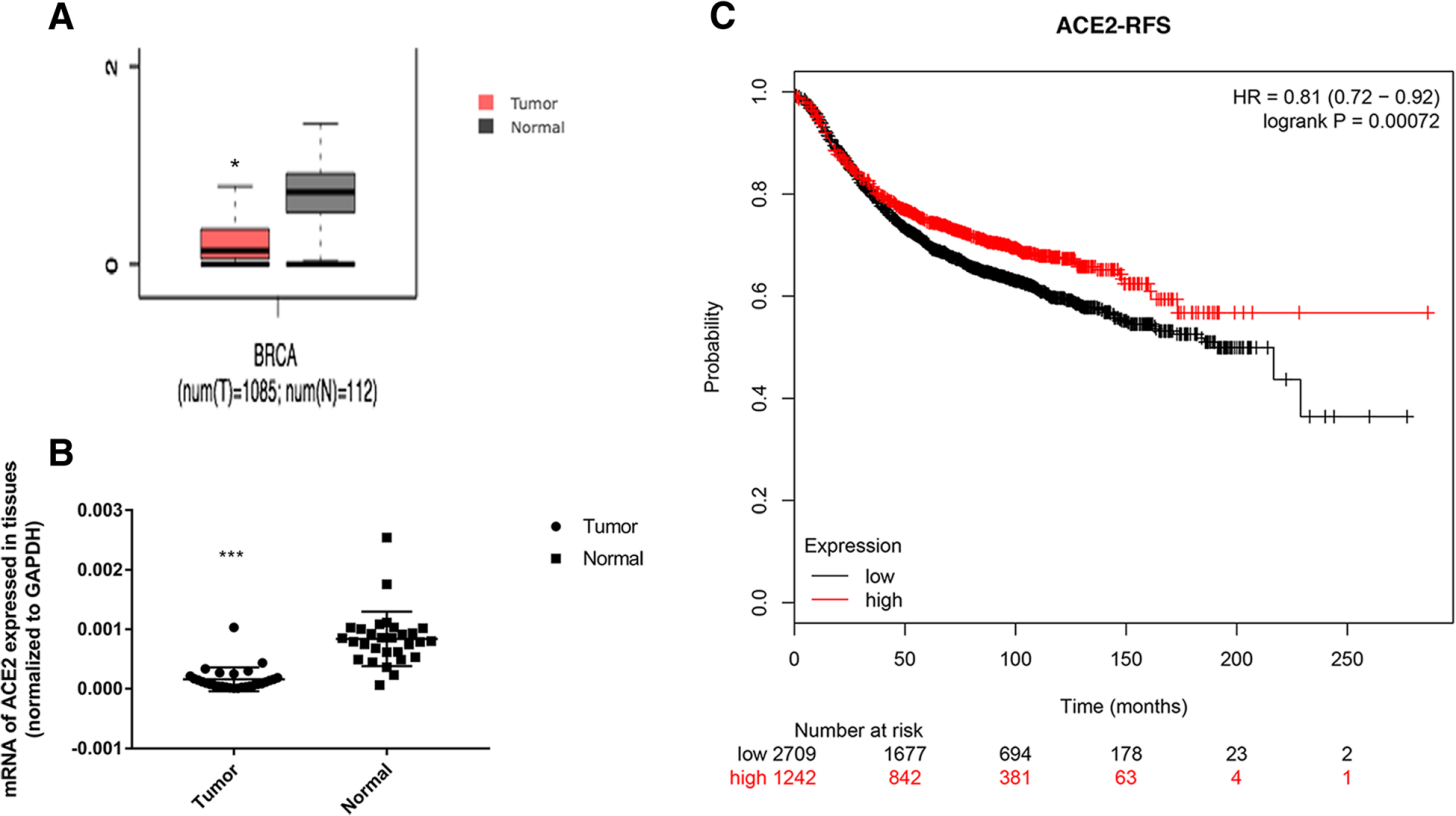
ACE2 is downregulated in breast cancer tissue, and this downregulation is associated with worse prognosis. (**a**) Expression of ACE2 in breast cancer and normal tissues in the GEPIA database (*n* = 1197). (**b**) Expression of ACE2 mRNA in breast cancer and paired normal tissues (*n* = 29). (**c**) Kaplan-Meier curve of RFS stratified by the ACE2 level using the GEO database (*n* = 3951)

To evaluate the role of ACE2 in breast cancer, we first analysed the expression of ACE2 in breast cancer and normal tissues using the GEPIA database (*n* = 1197). The level of ACE2 in breast cancer tumour tissue was significantly lower than that in normal tissue (Fig. 1a). We subsequently detected the mRNA expression of ACE2 in breast cancer and paired normal tissues from 29 breast cancer patients. In all the patients, the expression of ACE2 in tumour tissue was obviously lower than that in normal tissue, which was consistent with the GEPIA results (Fig. 1b). The clinical and pathological characteristics of the 29 breast cancer patients were separated into two groups according to the median ACE2 expression value (Additional file 2: Table S1). Additionally, further clinical and pathological data stratified by the ACE2 level were analysed using the TCGA database (*n* = 1174; median cut-off; Additional file 3: Table S2). This indicated that ACE2 might act as an anti-tumour factor in breast cancer. We subsequently constructed the Kaplan-Meier curve of breast cancer using the GEO database (*n* = 3951, best cut-off), which showed that a higher ACE2 level was associated with a longer RFS (Fig. 1c). These results revealed that ACE2 might play a favourable role in breast cancer progression.

### ACE2 inhibits breast cancer cell migration

**Fig. 2:**
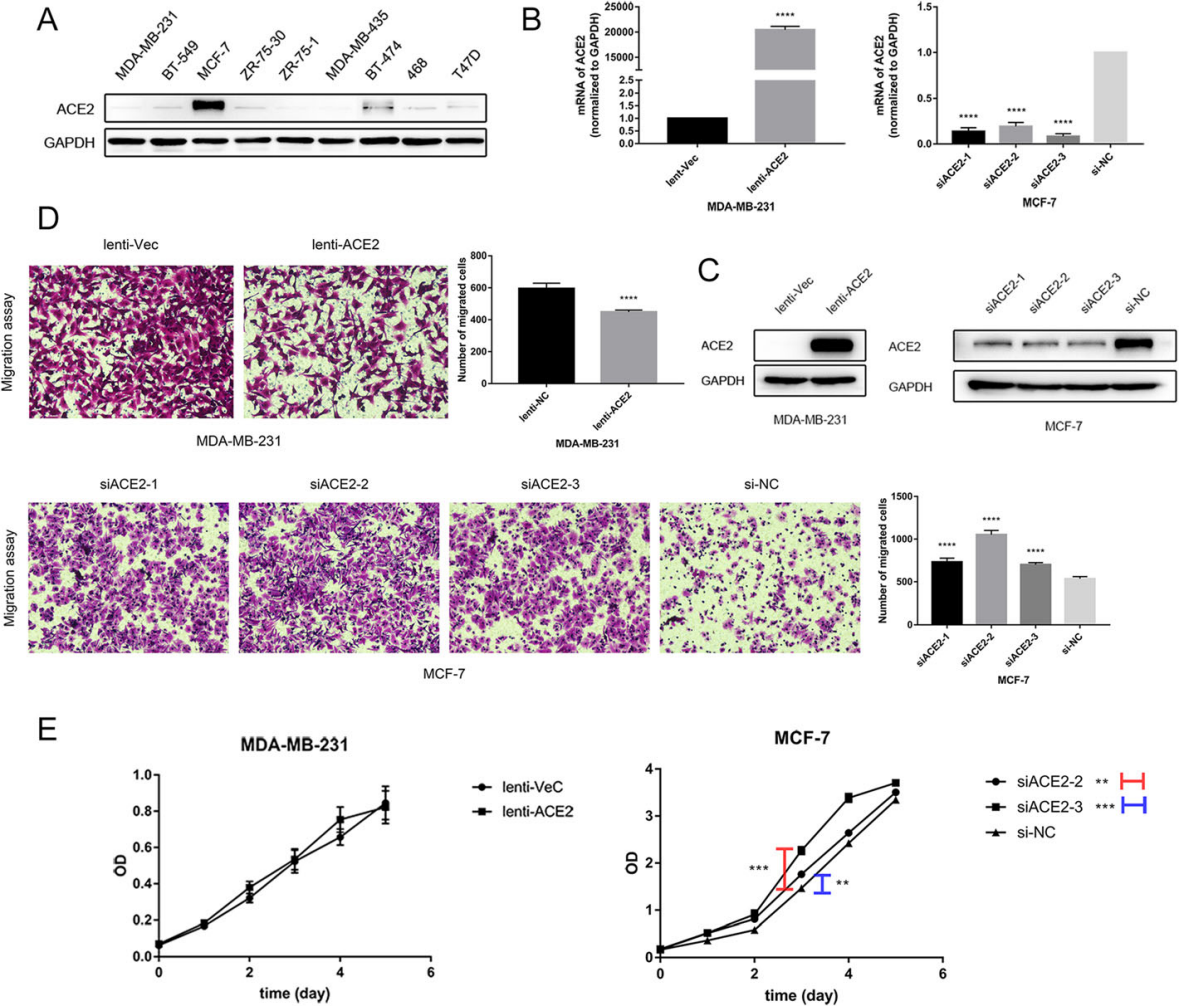
ACE2 inhibits breast cancer cell migration. (**a**) Basal expression of ACE2 in breast cancer cell lines. (**b**) mRNA level of ACE2 in MDA-MB-231 cells overexpressing ACE2 and ACE2-knockdown MCF-7 cells. (**c**) Protein levels of ACE2 in MDA-MB-231 cells overexpressing ACE2 and ACE2-knockdown MCF-7 cells. (**d**) Transwell migration assay of transfected MDA-MB-231 and MCF-7 cells. (**e**) CCK8 proliferation assay of transfected MDA-MB-231 and MCF-7 cells. Microscope magnification: 50×. *P < 0.05; **P < 0.01; ***P < 0.001; ****P < 0.0001

To clarify the role of ACE2 in breast cancer progression, we examined the expression of ACE2 in different breast cancer cell lines (MDA-MB-231, BT-549, MCF-7, ZR-75-30, ZR-75-1, MDA-MB-435, BT-474, 468, and T47D). A lower level of ACE2 was observed in the breast cancer cell lines with higher metastatic potency, such as MDA-MB-231 cells, whereas a higher expression of ACE2 was detected in the cell lines with weaker metastatic ability, such as MCF-7 cells (Fig. 2a). We subsequently established MDA-MB-231 cell lines stably expressing ACE2 (231-lenti-ACE2) and the control cell lines (231-lenti-Vec). Meanwhile, ACE2 was efficiently knocked down in MCF-7 cells by transfection using three different siRNA sequences (MCF-7-siACE2–1, MCF-7-siACE2–2, and MCF-7-siACE2–3) compared with the expression of ACE2 in the negative control cells (MCF-7-si-NC). The mRNA and protein levels of ACE2 expression in the transfected breast cancer cells were altered as expected (Fig. 2b and c). To estimate the effect of ACE2 on breast cancer cells, we conducted the Transwell migration assay and found that the migration of MDA-MB-231 cells overexpressing ACE2 was decreased compared with that of the control cells. While in MCF-7 cells, the migration ability was enhanced after the knockdown of ACE2 compared with that of the negative control (Fig. 2d). We also performed the CCK8 proliferation assay and found that ACE2 inhibited the proliferation of MCF-7 cells, whereas ACE2 had no effect on the proliferation of MDA-MB-231 cells (Fig. 2e). Thus, ACE2 might inhibit the development of breast cancer in vitro.

### ACE2 inhibits breast cancer angiogenesis in vitro

**Fig. 3:**
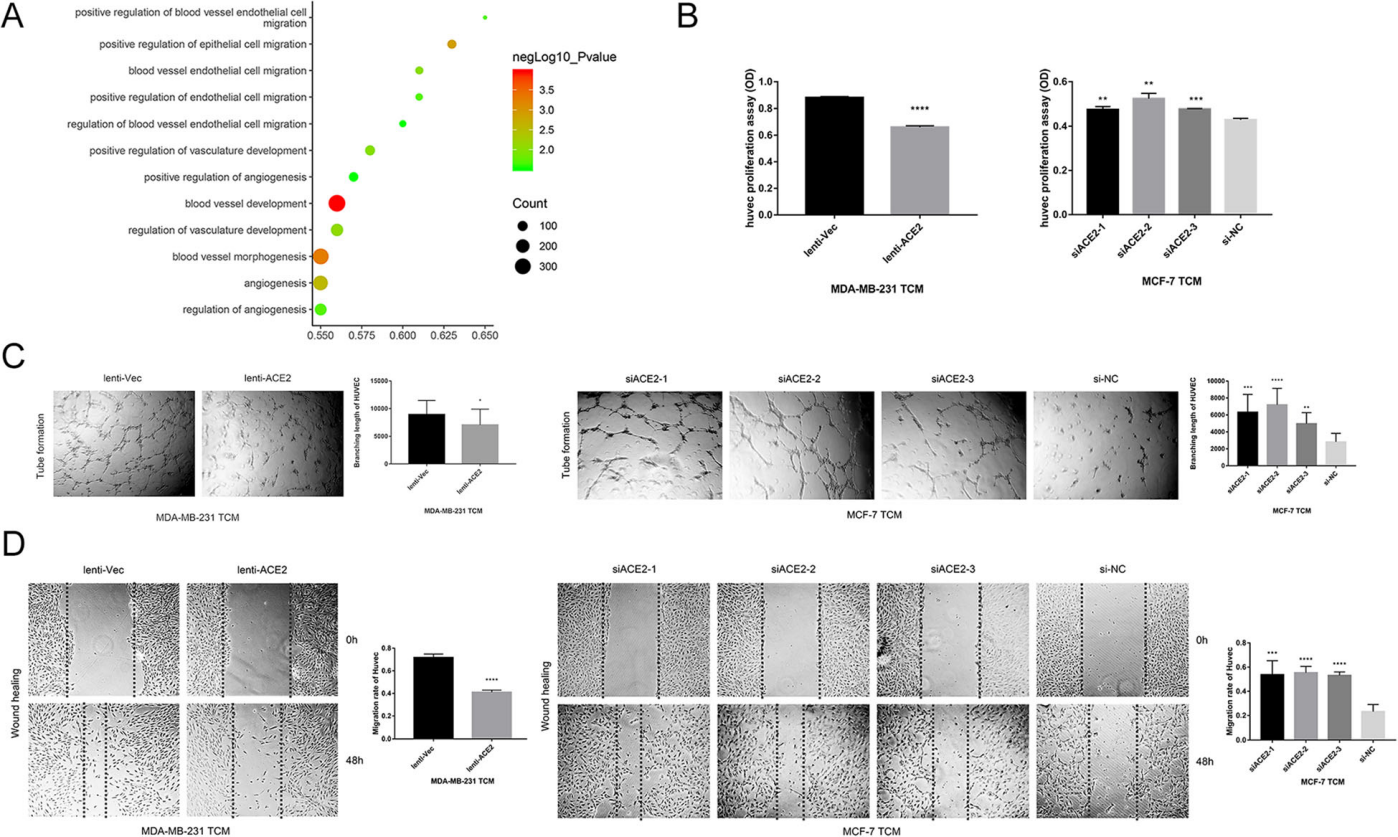
ACE2 inhibits breast cancer angiogenesis in vitro. (**a**) Bubble plot of ACE2 and breast cancer angiogenesis obtained from the GO enrichment analysis. (**b**) Proliferation of HUVECs after 72 h in the TCM from transfected MDA-MB-231 and MCF-7 cells. (**c**) Tube formation ability of HUVECs cultivated for 6 h in the TCM from transfected MDA-MB-231 and MCF-7 cells. (**d**) Wound healing ability of HUVECs after 48 h in the TCM from transfected MDA-MB-231 and MCF-7 cells. Microscope magnification: 50×. *P < 0.05; **P < 0.01; ***P < 0.001; ****P < 0.0001

Because ACE2 was reported to have a negative correlation with angiogenesis in HCC [37] and NSCLC [45], we further explored whether ACE2 inhibits breast cancer angiogenesis to prevent breast cancer progression. We performed GO enrichment of ACE2 in breast cancer first and found that ACE2 was obviously related to angiogenesis in breast cancer (Fig. 3a). To validate the results of the GO enrichment analysis, which showed that ACE2 influenced breast cancer angiogenesis, we then collected the TCM from the above-transfected breast cancer cells and performed HUVEC proliferation, tube formation and wound healing assays. The proliferation ability of HUVECs in the TCM from 231-lenti-ACE2 cells was decreased compared with that in the TCM from 231-lenti-Vec cells (Fig. 3b). However, the proliferation of HUVECs in the TCM from ACE2-knockdown MCF-7 cells was increased compared with that in the TCM from MCF-7-negative control cells (Fig. 2b). Additionally, the TCM from 231-lenti-ACE2 cells induced HUVECs to develop fewer and smaller tubes than the TCM from 231-lenti-Vec (Fig. 3c). Additionally, increased tube formation was obtained with HUVECs that were cultivated in the TCM from ACE2-knockdown MCF-7 cells compared with those that grew in the TCM from MCF-7-si-NC cells (Fig. 3c). Moreover, the migration rate of HUVECs in the TCM from 231-lenti-ACE2 cells was decreased compared with that in the TCM from 231-lenti-Vec cells (Fig. 3d). By contrast, HUVECs migrated for a longer distance in the TCM extracted from ACE2-knockdown MCF-7 cells than in the TCM from negative control cells (Fig. 3d). These results revealed that ACE2 inhibited breast cancer angiogenesis in vitro.

### ACE2 inhibits breast cancer metastasis and angiogenesis in a zebrafish model

**Fig. 4:**
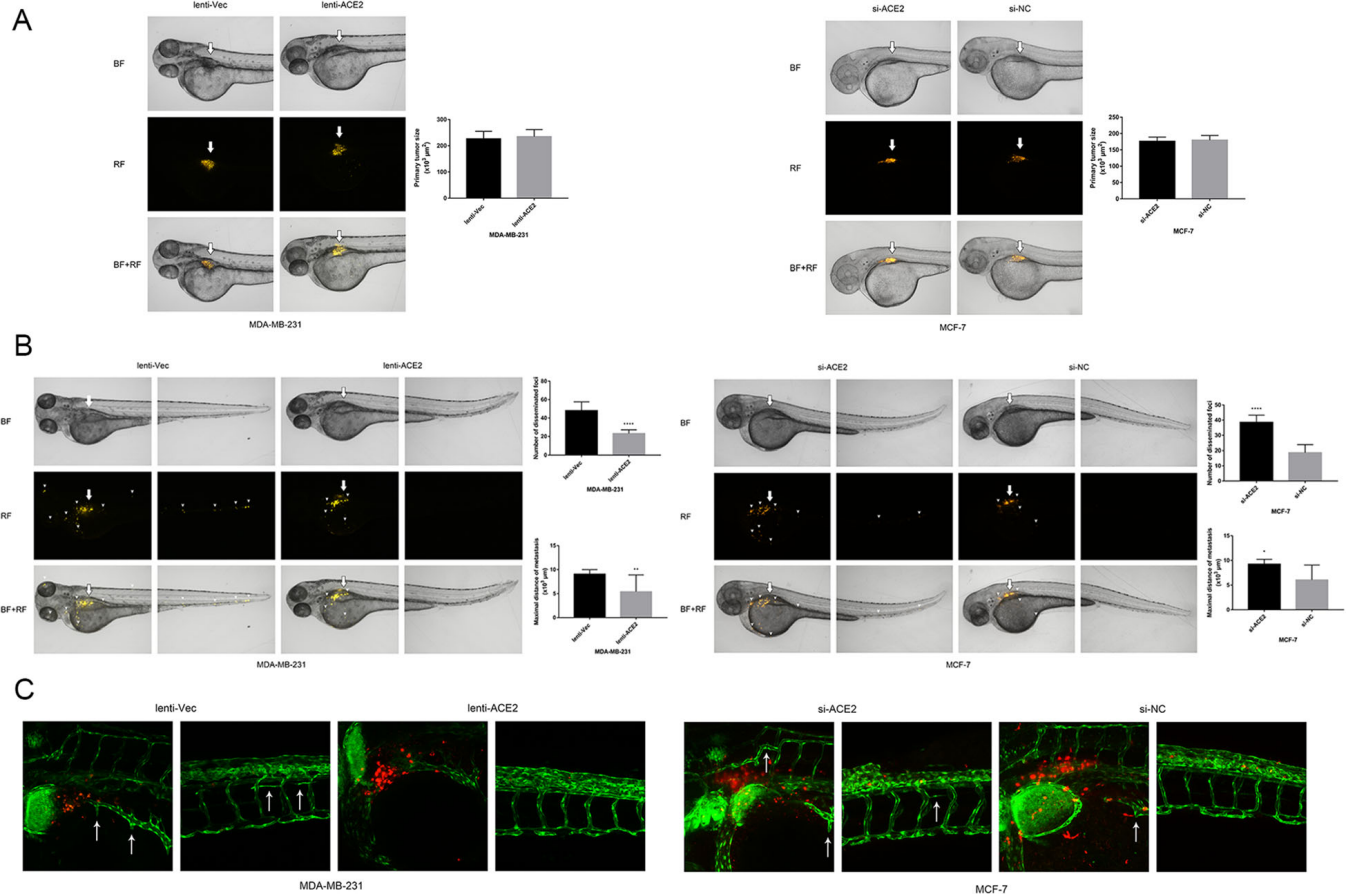
ACE2 inhibits breast cancer angiogenesis in vivo. (**a**) Equal number of Dil-labelled transfected MDA-MB-231 and MCF-7 cells were microinjected into the perivitelline space of embryos at 48 h post-fertilization. Thick arrows point to the injection sites. BF: bright field; RF: rhodamine fluorescence. (**b**) Dissemination and metastasis of the tumour cells in the zebrafish at 24 h post-injection (observed under a fluorescence microscope) and quantification of the number of disseminated foci and maximal metastatic distance. Small arrowheads point to the disseminated and metastatic tumour foci (MDA-MB-231, *n* = 9; MCF-7, *n* = 6). (**c**) Vasculature and neo-angiogenesis surrounding the primary tumour and metastasis sites in the zebrafish at 24 h post-injection (observed under a confocal microscope). Thin arrows point to newly formed tumour vessels. Fluorescence microscope magnification: 50×. Confocal microscope magnification: 200×. *P < 0.05; **P < 0.01; ***P < 0.001; ****P < 0.0001

Next, we established a zebrafish model to explore the effects of ACE2 on breast cancer metastasis and angiogenesis in vivo. The Dil-labelled 231-lenti-Vec and 231-lenti-ACE2 cells (stained in orange colour) were microinjected into the perivitelline space of embryos at 48 h post-fertilization, and the tumour sizes of the two groups of breast cancer cells were even (Fig. 4a). Additionally, equal numbers of the Dil-labelled MCF-7-siACE2–1 and MCF-7-si-NC cells were microinjected (Fig. 4a). The metastasis of the breast cancer cells in the zebrafish was then observed under a fluorescence microscope. Compared with 231-lenti-Vec cells, the 231-lenti-ACE2 cells resulted in fewer disseminated tumour foci and a shorter metastasis distance (Fig. 4b). Conversely, the ACE2-knockdown MCF-7 cells resulted in a larger number of disseminated tumour foci and a longer metastatic distance than the MCF-7-negative control cells (Fig. 4b).

Furthermore, using a confocal microscope, we observed the degree of angiogenesis around the primary tumour and metastasis sites. Because the vessels of the Tg (fli1: EGFP) zebrafish could glow with a green fluorescence, we found that the number of new formed twisty tumour vessels in the zebrafish injected with 231-lenti-ACE2 cells was less than that found in the zebrafish injected with 231-lenti-Vec cells (Fig. 4c). By contrast, the analysis of the primary tumour and metastasis sites in the zebrafish with MCF-7-siACE2 cells showed the development of more rugged tumour vessels than those found in the zebrafish with MCF-7-si-NC cells (Fig. 4c). Accordingly, the results indicated that ACE2 inhibited breast cancer metastasis and angiogenesis in vivo.

### ACE2 inhibits the VEGFa/VEGFR2/ERK pathway to suppress breast cancer angiogenesis

**Fig. 5:**
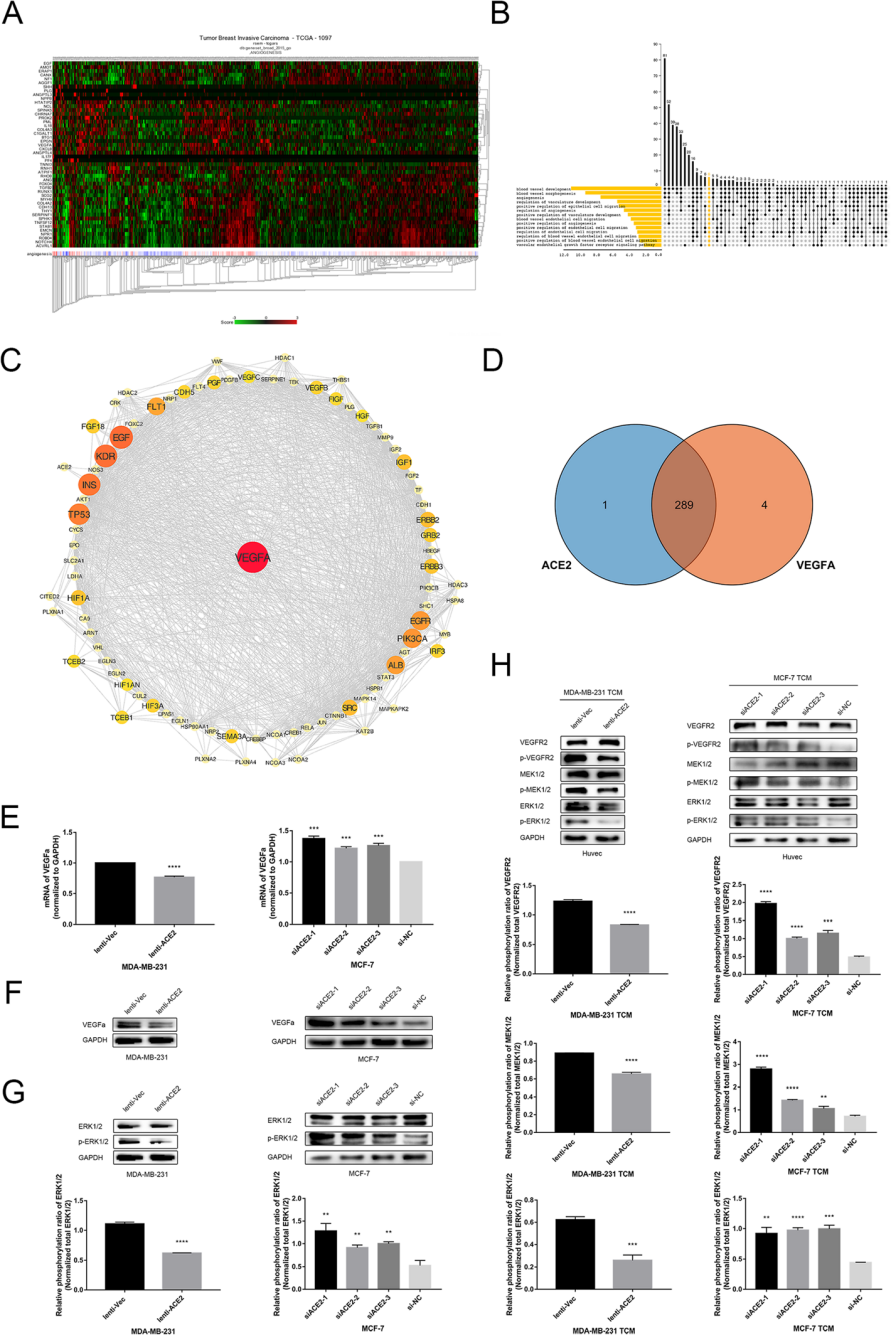
ACE2 inhibits the VEGFa/VEGFR2/ERK pathway to suppress breast cancer angiogenesis. (**a**) Heat map of the correlation of ACE2 with genes participating in breast cancer angiogenesis. (**b**) UpSet plot of the intersection of angiogenetic cytokines and ACE2 in breast cancer. (**c**) PPI plot of the correlation of ACE2 and potentially related genes. (**d**) KEGG pathway enrichment of ACE2 and VEGFa. (**e**) mRNA level of VEGFa in transfected MDA-MB-231 and MCF-7 cells. (**f**) Protein levels of VEGFa in transfected MDA-MB-231 and MCF-7 cells. (**g**) Phosphorylation level of ERK1/2 in transfected MDA-MB-231 and MCF-7 cells determined by Western blot analysis. (**h**) Western blot analysis of the phosphorylation level of VEGFR2, MEK1/2, and ERK1/2 in HUVECs cultivated for 24 h in the TCM of the transfected tumour cells. *P < 0.05; **P < 0.01; ***P < 0.001; ****P < 0.0001

To explore the mechanism through which ACE2 inhibits breast cancer angiogenesis, we analysed the correlation of ACE2 with genes that potentially participate in breast cancer angiogenesis using the TCGA database (Fig. 5a). Based on the intersection of the genes in the heat map, we identified six genes highly enriched in the GO term *angiogenesis* (Fig. 5b). We then calculated the rank of the hub genes using the STRING database and the Cytoscape tool cytoHubba and identified VEGFa as the most plausible mediator of ACE2 and the inhibition of breast cancer angiogenesis (Fig. 5c, Additional file 4: Table S3). Further KEGG pathway analysis revealed 289 pathways that might potentially mediate ACE2 and VEGFa (Fig. 5d, Additional file 5: Table S4). Thus, the findings suggested that VEGFa played a role in the anti-angiogenetic effect of ACE2 in breast cancer.

To confirm the results obtained with the databases, we detected the expression of VEGFa in MDA-MB-231 cells overexpressing ACE2 and ACE2- knockdown MCF-7 cells. Consistent with the results of the database analysis, the mRNA and protein levels of VEGFa were declined in the 231-lenti-ACE2 cells compared with the 231-lenti-Vec cells (Fig. 5e and f). Additionally, the expression of VEGFa was upregulated at both the mRNA and protein levels in the ACE2-knockdown MCF-7 cells compared with the MCF7-siNC cells (Fig. 5e and f). This indicated that VEGFa participated in the ACE2-mediated inhibition of breast cancer angiogenesis, and the underlying mechanism was studied further. It has been reported that extracellular signal-regulated kinase (ERK) signalling is a crucial pathway for the regulation of cell proliferation, differentiation, survival and cell motility in both normal and cancer cells [46–48], and the activation of ERK1/2 stimulates the expression of VEGFa in breast cancer cells [49]. Hence, we detected the phosphorylation level of ERK1/2 in transfected breast cancer cells and found that was decreased in the 231-lenti-ACE2 cells compared with the 231-lenti-Vec cells. However, the phosphorylation level of ERK1/2 in MCF-7-siACE2 cells was increased compared with that in the negative control cells (Fig. 5g). This revealed that ERK signalling played a role in the process through which ACE2 downregulated VEGFa expression in breast cancer cells.

It has been reported that secreted VEGFa usually combines with VEGFR2 on the endothelial cell membrane to promote angiogenesis [7]. Thus, we cultivated HUVECs in TCM from transfected breast cancer cells and then examined the related protein level of the incubated HUVECs. Accordingly, the phosphorylation level of VEGFR2 in HUVECs cultivated in the TCM from 231-lenti-ACE2 cells was decreased compared with that obtained in the TCM from 231-lenti-Vec cells. However, the TCM extracted from MCF-7-siACE2 cells stimulated the phosphorylation of VEGFR2 in HUVECs compared with that obtained with the TCM from negative control cells. It has been reported that the phosphorylation of VEGFR2 can further activate ERK signalling in endothelial cells. In this signalling process, mitogen-activated protein kinases 1 and 2 (MEK1/2) are phosphorylated and activated, which in turn phosphorylate and activate ERK1/2 [50, 51]. The phosphorylated ERK1/2 is subsequently translocated into the nucleus or activates other kinases and transcription factors to induce cell proliferation, migration, invasion and differentiation [52, 53]. Hence, we detected the phosphorylation levels of MEK1/2 and ERK1/2 in TCM-cultivated HUVECs and found decreased levels in HUVECs cultivated in the TCM of 231-lenti-ACE2 cells compared with those in the cells grown in the TCM of 231-lenti-Vec cells. However, the TCM of MCF-7-siACE2 cells induced increased phosphorylation of the two factors compared with the TCM of MCF-7-siNC cells (Fig. 5h). This indicated that the ERK pathway might participate in the anti-angiogenetic effect of ACE2. Therefore, ACE2 inhibits breast cancer angiogenesis by the VEGFa/VEGFR2/ERK pathway.

## Discussion

Although ACE2 is a member of the RAS family, it also reportedly serves as a potential anti-tumour molecule in various malignant diseases, including lung cancer [33–35], prostate cancer [36] and HCC [37]. In our study, we found that the expression of ACE2 in breast cancer was lower than that in normal tissue, and a lower level of ACE2 in breast cancer patients is related to a worse prognosis, which suggests that ACE2 might play a favourable role in breast cancer. Additionally, in line with a previous study [54], ACE2 inhibited the migration of breast cancer cells while suppressing the proliferation of MCF-7 cells. These evidences suggest that ACE2 acts as a potential tumour suppressor in breast cancer and restrains breast cancer progression.

Angiogenesis, a pivotal process in malignant tumour progression [11, 12], is crucial for tumour metastasis and plays an essential role in breast cancer survival and prognosis [13]. Our data revealed the effect of ACE2 on breast cancer angiogenesis, the crucial step of metastasis. In vitro, ACE2 inhibited the ability of breast cancer cells to promote HUVEC proliferation, tube formation and migration. And in vivo, ACE2 decreased breast cancer cell-induced neo-angiogenesis in a zebrafish model. Additionally, ACE2 impaired the dissemination and metastasis of breast cancer cells in vivo, which was likely due to the ACE2-induced reduction in the number of tumour vessels and the ACE2-mediated deficiency in the tumour microenvironment. These results imply that ACE2, the metastasis inhibitor, might interrupt breast cancer angiogenesis, which, in turn, disturbs the process of metastasis. This anti-angiogenetic function of ACE2 in breast cancer is consistent with that observed in other malignant tumours. For example, in HCC, ACE2 is negatively correlated with CD34 [37], and in NSCLC, ACE2 suppresses angiogenesis and inhibits VEGFa expression [45].

**Fig. 6:**
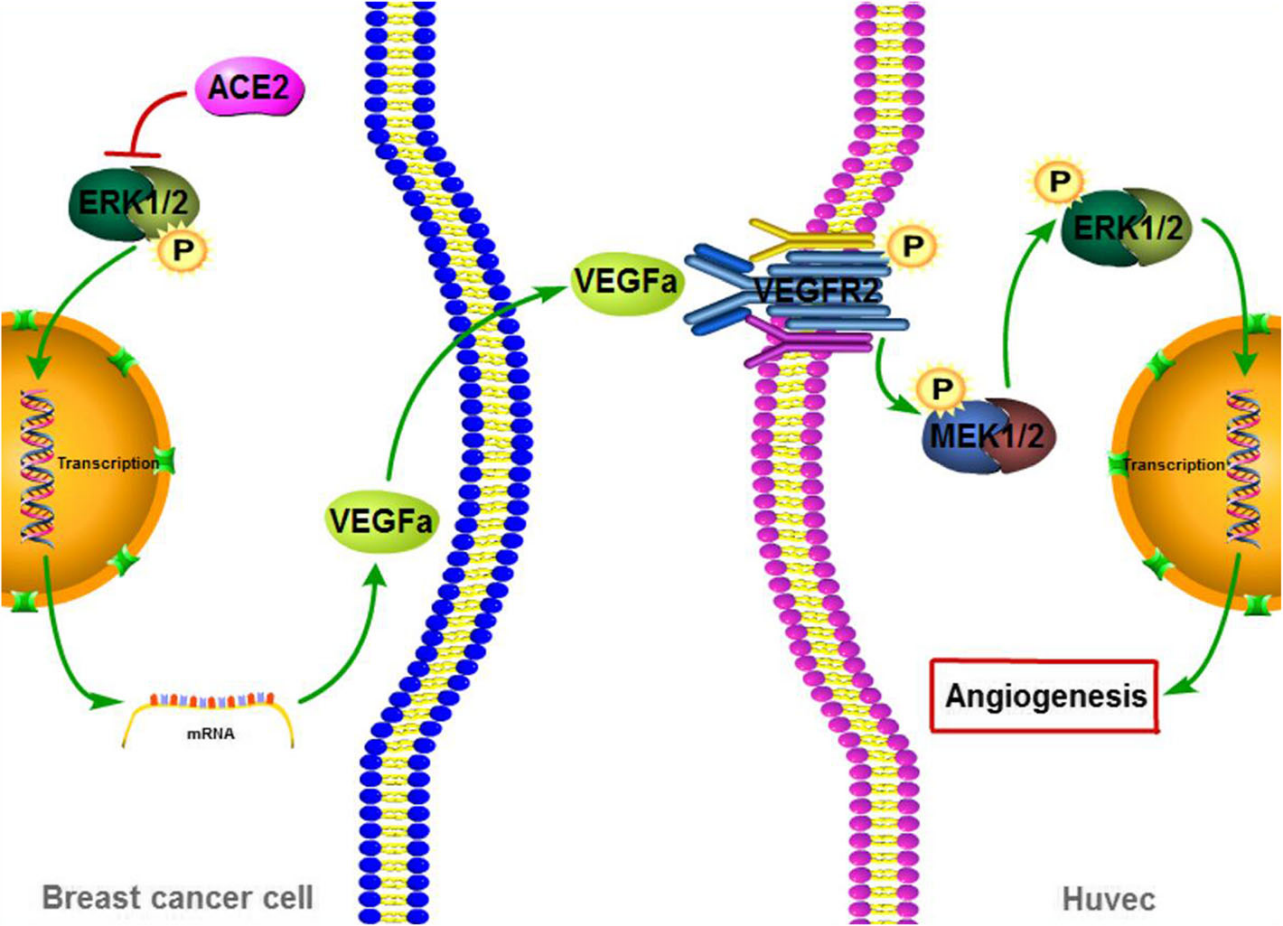
Schematic diagram summarizing the signalling pathway through which ACE2 inhibits breast cancer angiogenesis

Many pro-angiogenic factors mediate tumour angiogenesis, and among these, VEGFa might play a major role in the process through which ACE2 inhibits breast cancer angiogenesis, as demonstrated through our database analysis. Further qPCR and Western blotting results concordantly showed that ACE2 downregulated VEGFa expression in breast cancer cells, similar to the previous results in NSCLC [45]. Additionally, in our study, ACE2 inhibited the phosphorylation of ERK1/2 in breast cancer cells, which revealed that ERK signalling participated in the ACE2-mediated regulation of the expression of VEGFa. This is consistent with that obtained in a previous study, which showed that the ERK pathway modulated VEGFa [49]. It has been well recognized that VEGFa from tumour cells combines VEGFR2 on the membrane of neighbouring endothelial cells. This combination phosphorylates and activates VEGFR2, which further phosphorylates and activates the ERK signalling pathway. In the cascade reaction of the ERK pathway, MEK1/2 is phosphorylated and activated, which in turn phosphorylates and activates ERK1/2. This leads to the nuclear translocation or activation of other molecules to promote HUVEC proliferation, differentiation and migration, which finally facilitate angiogenesis [55]. In our study, ACE2 inhibited the phosphorylation of VEGFR2, MEK1/2, and ERK1/2 in HUVECs through the downregulation of VEGFa in breast cancer cells. Therefore, the VEGFa/VEGFR2/ERK pathway might be the mechanism by which ACE2 inhibits breast cancer angiogenesis and metastasis (Fig. 6).

Presently, anti-tumour drugs have already been demonstrated to target the VEGF pathway, and among these drugs, bevacizumab has been widely used clinically for the treatment of multiple cancers, particularly NSCLC [22]. However, this drug cannot significantly improve the survival of breast cancer patients, and the mechanisms underlying this unpleasant result remain controversial. However, ACE2, as an upstream mediator of VEGFa, could inhibit breast cancer angiogenesis and might thus be a promising therapeutic target to postpone the progression of breast cancer. To date, various ACE2 agonists have been designed for different diseases. However, the effects of ACE2 agonists on breast cancer angiogenesis remain unknown, and further study is needed.

## Conclusions

We explored the role of ACE2 in breast cancer angiogenesis and found that ACE2 could inhibit breast cancer angiogenesis and metastasis both in vitro and in vivo and suppress the VEGFa/VEGFR2/ERK pathway. This implied the potential therapeutic value of ACE2 in breast cancer.

## Additional files

Additional file 1: Supporting methods. (DOCX 19 kb)

Additional file 2: **Table S1.** Clinical and pathological characteristics of breast cancer patients. (DOCX 17 kb)

Additional file 3: **Table S2.** TCGA breast cancer clinical data stratified by the ACE2 expression level. (DOCX 20 kb)

Additional file 4: **Table S3.** Hub gene selection. (DOCX 19 kb)

Additional file 5: **Table S4.** KEGG pathways between ACE2 and VEGFa. (DOCX 32 kb)

## Abbreviations

ACE2: Angiotensin-converting enzyme 2
AngI: Angiotensin I
AngII: Angiotensin II
AT1R: AngII type 1 receptor
AT2R: AngII type 2 receptor
ATCC: American Type Culture Collection
BF: Bright field
DMEM: Dulbecco’s modified Eagle’s medium
ECM: Endothelial cell medium;
EMT: Epithelial-to-mesenchymal transition
FBS: Foetal bovine serum;
GEO: Gene Expression Omnibus
GEPIA: Gene Expression Profiling Interactive Analysis
GO: Gene Ontology
HCC: Hepatocellular carcinoma
HUVEC: Human umbilical vein endothelial cell
KEGG: Kyoto Encyclopedia of Genes and Genomes
MVD: Microvessel density
NSCLC: Non-small cell lung cancer
PVDF: Polyvinylidene fluoride film
qPCR: Reverse transcription-quantitative polymerase chain reaction
RAS: Renin-angiotensin system
RF: Rhodamine fluorescence
siRNA: small interfering RNA
TCGA: The Cancer Genome Atlas
TCM: Tumour conditioned medium
VEGFa: Vascular endothelial growth factor A
VEGFR2: Vascular endothelial growth factor receptor 2
ERK1/2: Extracellular signal-regulated protein kinase 1/2
MEK1/2: Mitogen-activated protein kinase 1/2
Ang(1–7): Angiotensin (1–7)
Ang(1–9): Angiotensin (1–9)
