# Supplementary material for: ACE2 inhibits breast cancer angiogenesis via suppressing the VEGFa/VEGFR2/ERK pathway
Source: J Exp Clin Cancer Res. 2019 Apr 25;38:173. doi: 10.1186/s13046-019-1156-5 (PMC6482513; doi:10.1186/s13046-019-1156-5)
Supplement: Supplementary file 1 — Supporting methods. (DOCX 19 kb) [file 13046_2019_1156_MOESM1_ESM.docx]

**Supporting Methods**

***Cell transfection***

The sequence of the pCMV-ACE2-EGFP-puro plasmid in the lentivirus was as follows:

TTTGTTAGACGAAGCTTGGGCTGCAGGTCGACTCTAGAGGATCCCCGGGTACCGGTCGCCACCATGGCGGAGCCGAGCGGCTCGCCCGTGCACGTCCAGCTTCCCCAGCAGGCGGCCCCGGTGACAGCGGCGGCGGCGGCGGCCCCGGCGGCCGCGACAGCAGCGCCGGCCCCGGCAGCTCCCGCGGCCCCGGCCCCGGCCCCGGCCCCGGCGGCACAGGCTGTCGGCTGGCCCATCTGCAGGGACGCGTACGAGCTGCAGGAGGTTATCGGCAGTGGAGCTACTGCTGTGGTTCAGGCAGCCCTATGCAAACCCAGGCAAGAACGTGTAGCAATAAAACGGATCAACTTGGAAAAATGCCAGACCAGTATGGATGAACTATTAAAAGAAATTCAAGCCATGAGTCAGTGCAGCCATCCCAACGTAGTGACCTATTACACCTCTTTTGTGGTCAAAGATGAACTTTGGCTGGTCATGAAATTACTAAGTGGAGGTTCAATGTTGGATATCATAAAATACATTGTCAACCGAGGAGAACACAAGAATGGAGTTCTGGAAGAGGCAATAATAGCAACAATTCTTAAAGAGGTTTTGGAAGGCTTAGACTATCTACACAGAAACGGTCAGATTCACAGGGATTTGAAAGCTGGTAATATTCTTCTGGGTGAGGATGGTTCAGTACAAATAGCAGATTTTGGGGTAAGTGCGTTCCTAGCAACAGGGGGTGATGTTACCCGAAATAAAGTAAGAAAAACATTCGTTGGCACCCCATGTTGGATGGCTCCTGAAGTCATGGAACAGGTGAGAGGCTATGACTTCAAGGCTGACATGTGGAGTTTTGGAATAACTGCCATTGAATTAGCAACAGGAGCAGCGCCTTATCACAAATATCCTCCCATGAAAGTGTTAATGTTGACTTTGCAAAATGATCCACCCACTTTGGAAACAGGGGTAGAGGATAAAGAAATGATGAAAAAGTACGGCAAGTCCTTTAGAAAATTACTTTCACTGTGTCTTCAGAAAGATCCTTCCAAAAGGCCCACAGCAGCAGAACTTTTAAAATGCAAATTCTTCCAGAAAGCCAAGAACAGAGAGTACCTGATTGAGAAGCTGCTTACAAGAACACCAGACATAGCCCAAAGAGCCAAAAAGGTAAGAAGAGTTCCTGGGTCAAGTGGTCACCTTCATAAAACCGAAGACGGGGACTGGGAGTGGAGTGACGACGAGATGGATGAGAAGAGCGAAGAAGGGAAAGCAGCTTTTTCTCAGGAAAAGTCACGAAGAGTAAAAGAAGAAAATCCAGAGATTGCAGTGAGTGCCAGCACCATCCCCGAACAAATACAGTCCCTCTCTGTGCACGACTCTCAGGGCCCACCCAATGCTAATGAAGACTACAGAGAAGCTTCTTCTTGTGCCGTGAACCTCGTTTTGAGATTAAGAAACTCCAGAAAGGAACTTAATGACATACGATTTGAGTTTACTCCAGGAAGAGATACAGCAGATGGTGTATCTCAGGAGCTCTTCTCTGCTGGCTTGGTGGATGGTCACGATGTAGTTATAGTGGCTGCTAATTTACAGAAGATTGTAGATGATCCCAAAGCTTTAAAAACATTGACATTTAAGTTGGCTTCTGGCTGTGATGGGTCGGAGATTCCTGATGAAGTGAAGCTGATTGGGTTTGCTCAGTTGAGTGTCAGCCCGGTCGCCACCATGGTGAGCAAGGG

The sequences of the siRNAs used for the knockdown of ACE2 were as follows:

Sequence 1, CUGGGAUGCACAGAGAAUATT;

Sequence 2, GAGGAGACUAUGAAGUAAATT; and

Sequence 3, CCACACCUAAGCAUUUAAATT.

***RT-qPCR***

The temperature program for reverse transcription consisted of one cycle of 37 ℃ for 15 min, 85 ℃ for 5 s and 4 ℃ for 1 h, and the reaction comprised 10 µl of compound containing 1000 ng of RNA, 4 µl of RT and pure water.

The qPCR consisted of one cycle of 95℃ for 30 s, 50 cycles of 95 ℃ for 5 s and 60 ℃ for 30 s, one cycle of 95 ℃ for 5 s, 60 ℃ for 1 min and one cycle of 50 ℃ for 30 s. The 10-µl reaction comprised 1 µl of cDNA, 0.4 µl of the forward primer, 0.4 µl of the reverse primer, 5 µl of SYBR Green and 3.2 µl of pure water.

The sequences of the primers (Takara, Japan) were as follows:

GAPDH forward primer, GCACCGTCAAGGCTGAGAAC, reverse primer, TGGTGAAGACGCCAGTGGA;

ACE2 forward primer, CGAGTGGCTAATTTGAAACCAAGAA, reverse primer, ATTGATACGGCTCCGGGACA;

VEGF forward primer, GAGCCTTGCCTTGCTGCTCTA, reverse primer, CACCAGGGTCTCGATTGGATG; and

VEGFR2 forward primer, AGCCAGCTCTGGATTTGTGGA, reverse primer, CATGCCCTTAGCCACTTGGAA.

***Western blotting***

The antibodies used in the Western blot assay were as follows:

| Antibody | Company | Lot. number | Dilution |
| --- | --- | --- | --- |
| GAPDH | CST | 2118s | 1:2000 |
| ACE2 | Abcam | ab108252 | 1:1000 |
| VEGFa | Abcam | ab1316 | 1:100 |
| VEGFR2 | CST | 9698s | 1:1000 |
| p-VEGFR2 (Y1175) | CST | 2478s | 1:1000 |
| ERK | CST | 4695s | 1:1000 |
| p-ERK (T202/Y204) | CST | 4370p | 1:2000 |
| MEK | CST | 9122s | 1:1000 |
| p-MEK (S217/221) | CST | 9154p | 1:1000 |
