# Supplementary material for: ACE2 inhibits breast cancer angiogenesis via suppressing the VEGFa/VEGFR2/ERK pathway
Source: J Exp Clin Cancer Res. 2019 Apr 25;38:173. doi: 10.1186/s13046-019-1156-5 (PMC6482513; doi:10.1186/s13046-019-1156-5)
Supplement: Supplementary file 2 — Table S1. Clinical and pathological characteristics of breast cancer patients. (DOCX 17 kb) [file 13046_2019_1156_MOESM2_ESM.docx]

**Supporting table 1**

Clinical and pathological characteristics of breast cancer patients.

|  | Low ACE2 expression | High ACE2 expression | P value |
| --- | --- | --- | --- |
| Age |  |  | 0.362 |
| ≤50 y | 10(66.7%) | 7(50.0%) |  |
| >50 y | 5(33.3%) | 7(50.0%) |  |
| Pathologic grade |  |  | 0.915 |
| I | 2(14.3%) | 2(14.3%) |  |
| II | 7(50.0%) | 8(57.1%) |  |
| III | 5(35.7%) | 4(28.6%) |  |
| Pathologic stage |  |  | 0.984 |
| I | 6(40.0%) | 6(42.9%) |  |
| II | 8(53.3%) | 7(50.0%) |  |
| III | 1(6.7%) | 1(7.1%) |  |
| Tumour size |  |  | 0.119 |
| T1 | 12(80.0%) | 8(57.1%) |  |
| T2 | 2(13.3%) | 6(42.9%) |  |
| T3 | 1(6.7%) | 0(0.0%) |  |
| Lymph node |  |  | 0.092 |
| N0 | 8(53.3%) | 11(78.6%) |  |
| N1 | 7(46.7%) | 2(14.3%) |  |
| N2 | 0(0.0%) | 1(7.1%) |  |
| Metastasis |  |  | -- |
| No | 15(100.0%) | 14(100.0%) |  |
| Yes | 0(0.0%) | 0(0.0%) |  |
| ER |  |  | 0.498 |
| <1% | 1(6.7%) | 2(14.3%) |  |
| ≥1% | 14(93.3%) | 12(85.7%) |  |
| PR |  |  | 0.109 |
| <1% | 1(6.7%) | 4(28.6%) |  |
| ≥1% | 14(93.3%) | 10(71.4%) |  |
| Her2 |  |  | 0.941 |
| Negative | 13(86.7%) | 12(85.7%) |  |
| Positive | 2(13.3%) | 2(14.3%) |  |
| Ki-67 |  |  | 0.899 |
| <14% | 5(35.7%) | 4(33.3%) |  |
| ≥14% | 9(64.3%) | 8(66.7%) |  |

The median value of ACE2 mRNA expression was used as the cut-off value for high and low ACE2 expression.

ER: oestrogen receptor; PR: progesterone receptor; Her2: human epidermal growth factor receptor 2.
