# Supplementary material for: ACE2 inhibits breast cancer angiogenesis via suppressing the VEGFa/VEGFR2/ERK pathway
Source: J Exp Clin Cancer Res. 2019 Apr 25;38:173. doi: 10.1186/s13046-019-1156-5 (PMC6482513; doi:10.1186/s13046-019-1156-5)
Supplement: Supplementary file 3 — Table S2. TCGA breast cancer clinical data stratified by the ACE2 expression level. (DOCX 20 kb) [file 13046_2019_1156_MOESM3_ESM.docx]

**Supporting table 2**

TCGA breast cancer clinical data stratified by the ACE2 expression level.

|  | ACE2 expression level | |  |  |
| --- | --- | --- | --- | --- |
|  | Low (n=584) | High (n=590) | Total (n=1174) | P VALUE |
| Gender | | | | |
| FEMALE | 581 (99.5%) | 580 (98.3%) | 1161 (98.9%) |  |
| MALE | 3 (0.5%) | 10 (1.7%) | 13 (1.1%) | 0.098 |
| Age | | | | |
| Mean (SD) | 59.3 (13.1) | 58.3 (13.4) | 58.8 (13.3) |  |
| Median [min, max] | 60 [26, 90] | 57 [26, 90] | 59 [26, 90] |  |
| Stage | | | | |
| Stage I | 43 (7.4%) | 54 (9.2%) | 97 (8.3%) |  |
| Stage IA | 54 (9.2%) | 39 (6.6%) | 93 (7.9%) |  |
| Stage IB | 5 (0.9%) | 3 (0.5%) | 8 (0.7%) |  |
| Stage II | 2 (0.3%) | 4 (0.7%) | 6 (0.5%) |  |
| Stage IIA | 192 (32.9%) | 196 (33.2%) | 388 (33.0%) |  |
| Stage IIB | 128 (21.9%) | 138 (23.4%) | 266 (22.7%) |  |
| Stage III | 2 (0.3%) | NA | 2 (0.2%) |  |
| Stage IIIA | 80 (13.7%) | 88 (14.9%) | 168 (14.3%) |  |
| Stage IIIB | 14 (2.4%) | 14 (2.4%) | 28 (2.4%) |  |
| Stage IIIC | 43 (7.4%) | 27 (4.6%) | 70 (6.0%) |  |
| Stage IV | 7 (1.2%) | 14 (2.4%) | 21 (1.8%) |  |
| Stage X | 8 (1.4%) | 8 (1.4%) | 16 (1.4%) | 0.316 |
| Vital status | | | | |
| Alive | 537 (92.0%) | 525 (89.0%) | 1062 (90.5%) |  |
| Dead | 47 (8.0%) | 65 (11.0%) | 112 (9.5%) | 0.103 |
| Race | | | | |
| Asian | 21 (3.6%) | 41 (6.9%) | 62 (5.3%) |  |
| Black or African American | 99 (17.0%) | 102 (17.3%) | 201 (17.1%) |  |
| White | 398 (68.2%) | 403 (68.3%) | 801 (68.2%) |  |
| American Indian or Alaska Native | NA | 1 (0.2%) | 1 (0.1%) | 0.055 |
| History of neoadjuvant treatment | | | | |
| No | 571 (97.8%) | 579 (98.1%) | 1150 (98.0%) |  |
| Yes | 11 (1.9%) | 10 (1.7%) | 21 (1.8%) | 0.978 |
| Histological type | | | | |
| Infiltrating carcinoma | 1 (0.2%) | NA | 1 (0.1%) |  |
| Infiltrating ductal carcinoma | 408 (69.9%) | 424 (71.9%) | 832 (70.9%) |  |
| Infiltrating lobular carcinoma | 119 (20.4%) | 108 (18.3%) | 227 (19.3%) |  |
| Medullary carcinoma | 1 (0.2%) | 5 (0.8%) | 6 (0.5%) |  |
| Metaplastic carcinoma | 5 (0.9%) | 5 (0.8%) | 10 (0.9%) |  |
| Mixed histology | 12 (2.1%) | 18 (3.1%) | 30 (2.6%) |  |
| Mucinous Carcinoma | 9 (1.5%) | 8 (1.4%) | 17 (1.4%) |  |
| Other specify | 28 (4.8%) | 22 (3.7%) | 50 (4.3%) | 0.49 |
| Menopause status | | | | |
| Indeterminate | 17 (2.9%) | 19 (3.2%) | 36 (3.1%) |  |
| Peri | 21 (3.6%) | 19 (3.2%) | 40 (3.4%) |  |
| Post | 396 (67.8%) | 368 (62.4%) | 764 (65.1%) |  |
| Pre | 108 (18.5%) | 128 (21.7%) | 236 (20.1%) | 0.412 |
| ER | | | | |
| Negative | 128 (21.9%) | 119 (20.2%) | 247 (21.0%) |  |
| Positive | 439 (75.2%) | 436 (73.9%) | 875 (74.5%) |  |
| Indeterminate | NA | 2 (0.3%) | 2 (0.2%) | 0.699 |
| PR | | | | |
| Indeterminate | 2 (0.3%) | 2 (0.3%) | 4 (0.3%) |  |
| Negative | 191 (32.7%) | 171 (29.0%) | 362 (30.8%) |  |
| Positive | 374 (64.0%) | 383 (64.9%) | 757 (64.5%) | 0.576 |
| HER2 | | | | |
| Equivocal | 106 (18.2%) | 96 (16.3%) | 202 (17.2%) |  |
| Indeterminate | 8 (1.4%) | 4 (0.7%) | 12 (1.0%) |  |
| Negative | 299 (51.2%) | 304 (51.5%) | 603 (51.4%) |  |
| Positive | 85 (14.6%) | 88 (14.9%) | 173 (14.7%) | 0.596 |
| Metastasis | | | | |
| NO | 207 (35.4%) | 201 (34.1%) | 408 (34.8%) |  |
| YES | 7 (1.2%) | 5 (0.8%) | 12 (1.0%) | 0.821 |
| New tumour events | | | | |
| NO | 252 (43.2%) | 268 (45.4%) | 520 (44.3%) |  |
| YES | 332 (56.8%) | 322 (54.6%) | 654 (55.7%) | 0.468 |

*: significant

The median ACE2 mRNA expression value was used as the cut-off value for high and low ACE2 expression.

ER: oestrogen receptor; PR: progesterone receptor; Her2: human epidermal growth factor receptor 2.
