# Supplementary material for: ACE2 inhibits breast cancer angiogenesis via suppressing the VEGFa/VEGFR2/ERK pathway
Source: J Exp Clin Cancer Res. 2019 Apr 25;38:173. doi: 10.1186/s13046-019-1156-5 (PMC6482513; doi:10.1186/s13046-019-1156-5)
Supplement: Supplementary file 4 — Table S3. Hub gene selection. (DOCX 19 kb) [file 13046_2019_1156_MOESM4_ESM.docx]

**Supporting table 3**

Hub gene selection.

| **RANK** | **MCC** | **DMNC** | **MNC** | **Deg** | **EPC** | **BN** | **EC** | **Clo** | **Rad** | **BC** | **Str** | **Clustering Coefficient** |
| --- | --- | --- | --- | --- | --- | --- | --- | --- | --- | --- | --- | --- |
| 1 | VEGFA | IGF1 | VEGFA | VEGFA | VEGFA | ALB | ALB | VEGFA | VEGFA | VEGFA | HIF1A | FGF18 |
| 2 | EGF | GRB2 | HIF1A | HIF1A | HIF1A | SRC | SRC | HIF1A | HIF1A | HIF1A | VEGFA | SEMA3A |
| 3 | EGFR | PGF | KDR | KDR | EGF | HIF3A | VEGFA | KDR | KDR | INS | INS | IRF3 |
| 4 | KDR | ERBB3 | EGF | EGF | KDR | VEGFA | HIF1A | INS | INS | KDR | KDR | ERBB3 |
| 5 | IGF1 | ERBB2 | EGFR | EGFR | TP53 | HIF1A | INS | EGF | EGF | PIK3CA | PIK3CA | TCEB1 |
| 6 | FLT1 | FIGF | TP53 | TP53 | FLT1 | INS | ERBB2 | TP53 | TP53 | TP53 | TP53 | VEGFB |
| 7 | TP53 | CDH5 | PIK3CA | PIK3CA | INS | ERBB2 | EGF | PIK3CA | PIK3CA | FLT1 | FLT1 | TCEB2 |
| 8 | PIK3CA | VEGFB | FLT1 | INS | PIK3CA | EGF | TP53 | EGFR | EGFR | EGFR | EGFR | CDH5 |
| 9 | ERBB2 | VEGFC | ALB | FLT1 | EGFR | TP53 | FLT1 | ALB | ALB | EGF | EGF | HIF1AN |
| 10 | ALB | HGF | INS | ALB | ALB | FLT1 | PIK3CA | FLT1 | FLT1 | ALB | ALB | GRB2 |

Algorithm abbreviations: MCC, maximal clique centrality; DMNC, density of maximum neighbourhood component; MNC, maximum neighbourhood component; Deg, degree method; EPC, edge percolated component; BN, bottleneck; EC, eccentricity; Clo, closeness; Rad, radiality; BC, betweenness; and Str, stress.
