# Supplementary material for: ACE2 inhibits breast cancer angiogenesis via suppressing the VEGFa/VEGFR2/ERK pathway
Source: J Exp Clin Cancer Res. 2019 Apr 25;38:173. doi: 10.1186/s13046-019-1156-5 (PMC6482513; doi:10.1186/s13046-019-1156-5)
Supplement: Supplementary file 5 — Table S4. KEGG pathways between ACE2 and VEGFa. (DOCX 32 kb) [file 13046_2019_1156_MOESM5_ESM.docx]

**Supporting table 4**

KEGG pathways between ACE2 and VEGFa.

| No. KEGG pathway |
| --- |
| 1. Amphetamine addiction |
| 1. Rheumatoid arthritis |
| 1. Non alcoholic fatty liver disease (NAFLD) |
| 1. African trypanosomiasis |
| 1. Cocaine addiction |
| 1. Serotonergic synapse |
| 1. NF-kappa B signalling pathway |
| 1. Long-term depression |
| 1. Cell adhesion molecules (CAMs) |
| 1. Collecting duct acid secretion |
| 1. Ascorbate and aldarate metabolism |
| 1. Neurotrophin signalling pathway |
| 1. T cell receptor signalling pathway |
| 1. TGF beta signalling pathway |
| 1. Phosphatidylinositol signalling system |
| 1. Fatty acid biosynthesis |
| 1. Choline metabolism in cancer |
| 1. Tyrosine metabolism |
| 1. Ether lipid metabolism |
| 1. Thyroid hormone signalling pathway |
| 1. GnRH signalling pathway |
| 1. Glycosaminoglycan biosynthesis - chondroitin sulphate /dermatan sulphate |
| 1. Melanogenesis |
| 1. VEGF signalling pathway |
| 1. Glutathione metabolism |
| 1. Tuberculosis |
| 1. Steroid biosynthesis |
| 1. Peroxisome |
| 1. Complement and coagulation cascades |
| 1. DNA replication |
| 1. Influenza A |
| 1. Circadian rhythm |
| 1. Antigen processing and presentation |
| 1. Epstein Barr virus infection |
| 1. Legionellosis |
| 1. Phagosome |
| 1. Endocrine- and other factor- regulated calcium reabsorption |
| 1. Valine leucine and isoleucine biosynthesis |
| 1. Sphingolipid signalling pathway |
| 1. Adherens junction |
| 1. Folate biosynthesis |
| 1. Estrogen signalling pathway |
| 1. Toxoplasmosis |
| 1. Allograft rejection |
| 1. Renal cell carcinoma |
| 1. Hepatitis B |
| 1. Hepatitis C |
| 1. Focal adhesion |
| 1. Olfactory transduction |
| 1. Nitrogen metabolism |
| 1. Shigellosis |
| 1. Porphyrin and chlorophyll metabolism |
| 1. D Glutamine and D glutamate metabolism |
| 1. Riboflavin metabolism |
| 1. Neuroactive ligand receptor interaction |
| 1. HIF- 1 signalling pathway |
| 1. Dilated cardiomyopathy |
| 1. Fatty acid elongation |
| 1. Galactose metabolism |
| 1. Steroid hormone biosynthesis |
| 1. Gastric acid secretion |
| 1. Adrenergic signalling in cardiomyocytes |
| 1. Dopaminergic synapse |
| 1. Carbohydrate digestion and absorption |
| 1. Aminoacyl tRNA biosynthesis |
| 1. Starch and sucrose metabolism |
| 1. One carbon pool by folate |
| 1. Fc epsilon RI signalling pathway |
| 1. Pertussis |
| 1. Notch signalling pathway |
| 1. mRNA surveillance pathway |
| 1. Endocytosis |
| 1. Sulphur relay system |
| 1. Selenocompound metabolism |
| 1. Rap1 signalling pathway |
| 1. Propanoate metabolism |
| 1. Type II diabetes mellitus |
| 1. Acute myeloid leukaemia |
| 1. Oxytocin signalling pathway |
| 1. Pantothenate and CoA biosynthesis |
| 1. AMPK signalling pathway |
| 1. Viral carcinogenesis |
| 1. Nicotine addiction |
| 1. Glucagon signalling pathway |
| 1. Malaria |
| 1. Melanoma |
| 1. Leishmaniasis |
| 1. Central carbon metabolism in cancer |
| 1. Fatty acid degradation |
| 1. Endometrial cancer |
| 1. Pathogenic Escherichia coli infection |
| 1. Oxidative phosphorylation |
| 1. MAPK signalling pathway |
| 1. SNARE interactions in vesicular transport |
| 1. Ribosome biogenesis in eukaryotes |
| 1. Glutamatergic synapse |
| 1. Toll like receptor signalling pathway |
| 1. Bile secretion |
| 1. RNA transport |
| 1. Phenylalanine tyrosine and tryptophan biosynthesis |
| 1. Longevity regulating pathway in mammal |
| 1. Type I diabetes mellitus |
| 1. Salivary secretion |
| 1. Chemokine signalling pathway |
| 1. Butirosin and neomycin biosynthesis |
| 1. Ovarian steroidogenesis |
| 1. Vitamin B6 metabolism |
| 1. Chronic myeloid leukaemia |
| 1. Chagas disease (American trypanosomiasis) |
| 1. Pyruvate metabolism |
| 1. Measles |
| 1. Renin secretion |
| 1. Primary immunodeficiency |
| 1. HTLV I infection |
| 1. Amyotrophic lateral sclerosis (ALS) |
| 1. ABC transporters |
| 1. Non- small cell lung cancer |
| 1. beta- Alanine metabolism |
| 1. Prolactin signalling pathway |
| 1. Ubiquinone and other terpenoid quinone biosynthesis |
| 1. Mismatch repair |
| 1. ECM receptor interaction |
| 1. Dorso ventral axis formation |
| 1. Glycerophospholipid metabolism |
| 1. Spliceosome |
| 1. Other glycan degradation |
| 1. NOD- like receptor signalling pathway |
| 1. Homologous recombination |
| 1. Fructose and mannose metabolism |
| 1. Chemical carcinogenesis |
| 1. Glioma |
| 1. Metabolism of xenobiotics by cytochrome P450 |
| 1. Fat digestion and absorption |
| 1. Gap junction |
| 1. Cytosolic DNA sensing pathway |
| 1. Autoimmune thyroid disease |
| 1. Vitamin digestion and absorption |
| 1. Lysosome |
| 1. Caffeine metabolism |
| 1. PI3K Akt signalling pathway |
| 1. mTOR signalling pathway |
| 1. Cardiac muscle contraction |
| 1. Phospholipase D signalling pathway |
| 1. Tight junction |
| 1. Sulphur metabolism |
| 1. Pentose phosphate pathway |
| 1. Base excision repair |
| 1. Salmonella infection |
| 1. Proteoglycans in cancer |
| 1. Regulation of lipolysis in adipocytes |
| 1. Glycosylphosphatidylinositol (GPI) anchor biosynthesis |
| 1. Proximal tubule bicarbonate reclamation |
| 1. Linoleic acid metabolism |
| 1. Drug metabolism (cytochrome P450) |
| 1. Platelet activation |
| 1. Graft versus host disease |
| 1. Thyroid hormone synthesis |
| 1. RIG I- like receptor signalling pathway |
| 1. Asthma |
| 1. Wnt signalling pathway |
| 1. Taurine and hypotaurine metabolism |
| 1. Insulin resistance |
| 1. Vascular smooth muscle contraction |
| 1. Amoebiasis |
| 1. Parkinson’ s disease |
| 1. Regulation of autophagy |
| 1. Histidine metabolism |
| 1. Natural killer cell- mediated cytotoxicity |
| 1. Thiamine metabolism |
| 1. Sphingolipid metabolism |
| 1. Hypertrophic cardiomyopathy (HCM) |
| 1. Glycolysis - gluconeogenesis |
| 1. Epithelial cell signalling in Helicobacter pylori infection |
| 1. cGMP PKG signalling pathway |
| 1. Arginine and proline metabolism |
| 1. Morphine addiction |
| 1. Glycerolipid metabolism |
| 1. Basal transcription factors |
| 1. Transcriptional misregulation in cancer |
| 1. Tryptophan metabolism |
| 1. Hippo signalling pathway |
| 1. RNA polymerase |
| 1. Pancreatic cancer |
| 1. Glycosaminoglycan biosynthesis - keratan sulphate |
| 1. Aldosterone synthesis and secretion |
| 1. Valine, leucine and isoleucine degradation |
| 1. ErbB signalling pathway |
| 1. Cytokine- cytokine receptor interaction |
| 1. Basal cell carcinoma |
| 1. Purine metabolism |
| 1. Glycosphingolipid biosynthesis - ganglio series |
| 1. Axon guidance |
| 1. Nicotinate and nicotinamide metabolism |
| 1. Jak STAT signalling pathway |
| 1. B cell receptor signalling pathway |
| 1. Nucleotide excision repair |
| 1. Lysine biosynthesis |
| 1. Citrate cycle (TCA cycle) |
| 1. Taste transduction |
| 1. Lipoic acid metabolism |
| 1. GABAergic synapse |
| 1. Colorectal cancer |
| 1. Ubiquitin- mediated proteolysis |
| 1. Terpenoid backbone biosynthesis |
| 1. Retinol metabolism |
| 1. cAMP signalling pathway |
| 1. Staphylococcus aureus infection |
| 1. Alanine, aspartate and glutamate metabolism |
| 1. Progesterone- mediated oocyte maturation |
| 1. Arginine biosynthesis |
| 1. Aldosterone- regulated sodium reabsorption |
| 1. Non- homologous end joining |
| 1. Cysteine and methionine metabolism |
| 1. Glycine, serine and threonine metabolism |
| 1. Apoptosis |
| 1. Insulin secretion |
| 1. Synaptic vesicle cycle |
| 1. Calcium signalling pathway |
| 1. Leukocyte transendothelial migration |
| 1. Mineral absorption |
| 1. Lysine degradation |
| 1. Inositol phosphate metabolism |
| 1. Hedgehog signalling pathway |
| 1. Signalling pathways regulating the pluripotency of stem cells |
| 1. Thyroid cancer |
| 1. Vasopressin- regulated water reabsorption |
| 1. Circadian entrainment |
| 1. Protein digestion and absorption |
| 1. Proteasome |
| 1. Vibrio cholerae infection |
| 1. Small cell lung cancer |
| 1. Glycosaminoglycan biosynthesis - heparan sulphate /heparin |
| 1. p53 signalling pathway |
| 1. MicroRNAs in cancer |
| 1. Protein export |
| 1. Inflammatory bowel disease (IBD) |
| 1. Cholinergic synapse |
| 1. FoxO signalling pathway |
| 1. Amino sugar and nucleotide sugar metabolism |
| 1. Fanconi anaemia pathway |
| 1. Renin angiotensin system |
| 1. Biotin metabolism |
| 1. Phototransduction |
| 1. Long- term potentiation |
| 1. Haematopoietic cell lineage |
| 1. Maturity onset diabetes of the young |
| 1. Synthesis and degradation of ketone bodies |
| 1. Viral myocarditis |
| 1. Retrograde endocannabinoid signalling |
| 1. Primary bile acid biosynthesis |
| 1. Oocyte meiosis |
| 1. Ras signalling pathway |
| 1. Pyrimidine metabolism |
| 1. Protein processing in the endoplasmic reticulum |
| 1. Bladder cancer |
| 1. Ribosome |
| 1. Butanoate metabolism |
| 1. Cell cycle |
| 1. Regulation of actin cytoskeleton |
| 1. Other types of O glycan biosynthesis |
| 1. Bacterial invasion of epithelial cells |
| 1. Arrhythmogenic right ventricular cardiomyopathy (ARVC) |
| 1. Glycosphingolipid biosynthesis - globo series |
| 1. AGE RAGE signalling pathway in diabetic complications |
| 1. Prostate cancer |
| 1. RNA degradation |
| 1. Intestinal immune network for IgA production |
| 1. alpha Linolenic acid metabolism |
| 1. Glyoxylate and dicarboxylate metabolism |
| 1. Glycosphingolipid biosynthesis - lacto and neolacto series |
| 1. Pathways in cancer |
| 1. Phenylalanine metabolism |
| 1. Arachidonic acid metabolism |
| 1. Herpes simplex infection |
| 1. Alcoholism |
| 1. Inflammatory mediator regulation of TRP channels |
| 1. Mucin type O- glycan biosynthesis |
| 1. PPAR signalling pathway |
| 1. N- Glycan biosynthesis |
| 1. Huntington’ s disease |
| 1. Adipocytokine signalling pathway |
| 1. Prion diseases |
| 1. Fc gamma R- mediated phagocytosis |
| 1. Drug metabolism and other enzymes |
| 1. Biosynthesis of unsaturated fatty acids |
| 1. Alzheimer’ s disease |
| 1. TNF signalling pathway |
| 1. Pentose and glucuronate interconversions |
| 1. Glycosaminoglycan degradation |
